# Emotional Eating in Pregnant Women during the COVID-19 Pandemic and Its Association with Dietary Intake and Gestational Weight Gain

**DOI:** 10.3390/nu12082250

**Published:** 2020-07-28

**Authors:** Jian Zhang, Yumei Zhang, Shanshan Huo, Yidi Ma, Yalei Ke, Peiyu Wang, Ai Zhao

**Affiliations:** 1 Vanke School of Public Health, Tsinghua University, Beijing 100091, China; zhangjian92@pku.edu.cn; 2 School of Public Health, Peking University, Beijing 100191, China; yumeizhang_bjmu@163.com (Y.Z.); shanshanhuo_bjmu@163.com (S.H.); yidima_bjmu@163.com (Y.M.); yaleike_bjmu@163.com (Y.K.); peiyuwang_bjmu@163.com (P.W.)

**Keywords:** COVID-19, emotional eating, dietary intake, pregnant women, gestational weight gain

## Abstract

Reproductive health is a significant public health issue during pandemics; however, the impacts of the novel 2019 coronavirus disease (COVID-19) on noninfected pregnant women are still unknown. This study intends (1) to examine whether emotional eating (EE) occurred during the pandemic triggered by disease concerns and (2) to explore the associations among EE, dietary changes, and gestational weight gain (GWG). Based on an online survey, 640 new mothers who experienced the lockdown in their third trimester were recruited from seven provinces in China. EE was evaluated with the Chinese version of the Dutch Eating Behavior Questionnaire, EE domain. A self-designed e-questionnaire was used to collect the data of participants on the sociodemographic characteristics, concerns about the COVID-19 pandemic, maternity information, physical activities, and dietary changes during lockdown. The results show that the average EE score was 26.5 ± 8.3, and women living in a severely affected area, who are very worried about the pandemic and who had less physical activity had a higher tendency of EE. Although there is a dietary pattern changed during pandemic, the average GWG in the studied group was in the normal range. However, a higher EE score was associated with a significant excess of GWG in women not from Wuhan (EE score 33–65 vs. 13–22: adjusted Odd Ratio (OR), 95% Confidence Interval (CI) = 1.90, 1.08–3.32). The sensitivity analysis that additionally adjusted for the pregestational body mass index and gestational metabolic disease was consistent with this result. The mediation model was also examined and showed that, after adjusting for living area and exercise, EE was associated with significantly increased consumption of cereals (EE score 33–65 vs. 13–22: adjusted OR, 95% CI = 2.22, 1.29–3.82) and oil (EE score 33–65 vs. 13–22: adjusted OR, 95% CI = 3.03, 1.06–8.69) but decreased consumption of fish and seafood (EE score 33–65 vs. 13–22: adjusted OR, 95% CI = 1.88, 1.14–3.11; 23–32 vs. 13–22: adjusted OR, 95% CI = 1.79, 1.20–2.66). In conclusion, this study indicated that EE occurred in a proportional number of pregnant women during the COVID-19 pandemic and is associated with excess GWG mediated by increased intake of certain foods. The findings suggest the need for psychosocial and nutritional education and interventions during pregnancy checkups. Further studies are needed to determine modifiable psychosocial predictors and potential nutritional concerns in pregnant women during disease outbreaks.

## 1. Introduction

By 28 May 2020, the novel 2019 coronavirus disease (COVID-19) has reached 217 countries and has infected more than 349,000 confirmed cases [1,2]. Due to the highly contagious nature of this novel coronavirus, many countries have adopted several unprecedented measures to control disease transmission, including the suspension of public transportation, closure of public spaces, and isolation and management for infected people and suspected cases [3]. A “shelter at home” policy is also required or encouraged for the uninfected residents in numerous countries [3]. These measures more or less substantially changed people’s lifestyle.

Reproductive health is a significant public health issue during pandemics [4]; however, there is a limited amount of information on how the novel coronavirus affects pregnant women. Although several studies indicated that pregnant women are no more likely to be at a higher risk of becoming seriously ill from COVID-19 [5], a previous study revealed women experience changes in their bodies that may increase the risk of other illnesses, such as viral respiratory infections [6]. This uncertainty is feeding many pregnant women’s anxiety. As Rashidi Fakari and Simbar noted, several concerns regarding COVID-19 during pregnancy have been raised, including (i) the presence of family members given quarantine constraints; (ii) potential virus exposure during visits to physicians; (iii) potential requirement of early termination of pregnancy through elective cesarean section; and (iv) potential postpartum complications [7]. As a consequence, when a stressful situation of this magnitude arises, people often experience substantial changes in their behaviors, such as eating behaviors, which are described as emotional eating (EE) [8].

These emotion-based changes in eating behavior range from overeating or binge eating to severe caloric restriction [9]. EE has been seen in people after natural disasters, such as earthquake [10], but it has not yet been reported in the COVID-19 pandemic. However, psychologists have claimed that there is a high risk of occurrence of EE during the COVID-19 pandemic and that people should learn how to cope with it [11]. EE may cause impacts on pregnant women. On the one hand, excessive weight gain caused by excessive intake could lead to many adverse pregnancy outcomes, such as higher gestational weight gain (GWG), higher caesarean section rate, macrosomia, and early onset obesity [12]. On the other hand, insufficient food intake during pregnancy may also restrict intrauterine growth, may contribute to malnutrition in offspring, and may cause life-long effects [13]. In addition, regular pregnancy checks were more or less impacted by the pandemic, which put pregnant women at a higher risk of poor perinatal health management [4]. To the best of our knowledge, there is no such research exploring the effects of COVID-19 on noninfected pregnant women.

This study recruited new mothers who experienced self-isolation in their third trimester (from February to April) to explore the prevalence of EE during the COVID-19 pandemic, dietary intake, and its association with gestational weight gain.

## 2. Materials and Methods

### 2.1. Study Design and Participants

This study, which had a retrospective design, was conducted from 11 April to 25 May 2020. A multistage sampling method was used to collect data from new mothers, who just gave birth within 7 days. We initially purposely selected Wuhan, Hubei province, Beijing, and six other places according to their geographic location (Hebei, Henan, Jiangxi, Jiangsu, Guangxi, and Gansu). Secondly, we selected one or two hospitals in the urban areas of the above places with convenience. Then, every woman who gave birth in this period in the studied hospitals was invited by doctors and nurses to voluntarily participate in this survey by scanning the Quick Response (QR) code of the e-questionnaire via cellphone. The QR code was placed at the in-patient ward in every studied hospital. A total of 878 women participated in this survey. The inclusion criteria were (1) gave birth within the past 7 days; (2) full-term birth (≥37 w); (3) aged from 18 to 45 years; (4) lived in the investigated areas in the past three months; and (5) a single birth. The exclusion criteria were prior infection with COVID-19, a birth defect in the child, and severe disease (e.g., cancer, sever infection, or liver or kidney failure) during pregnancy or after labor. In addition, in the middle part of the e-questionnaire, one test prompt was used to identify whether the participants seriously responded to the questions: Please select the answer “rare” for this question. The women who answered other options except “rare” were considered as those who did not seriously respond to the questions and were excluded from this study (*N* = 88). Finally, data of 640 participants were involved in the analysis.

### 2.2. Data Collection

The e-questionnaire was conducted with Wenjuanxing (Wenjuan xing Tech Co. Ltd., Changsha, China), an online survey platform. The questionnaire includes five parts: sociodemographic characteristics, physical activity level, maternal medical history, EE during self-isolation time, and changes in dietary intake.

The sociodemographic characteristics include maternal age, family income, maternal education level, residency, and living areas. The data of confirmed infected cases in each investigated place (by 31 May) were obtained from the Distribution of COVID-19 Report on the website of the National Health Commission of the People’s Republic of China [14]. Whether participants knew someone confirmed with COVID-19 was investigated. In addition, a self-assessment on concerns about the COVID-19 situation was obtained; the women were asked to rate a 0–10 score according to their degree of concern (0 indicated not worried at all, and 10 indicated severely worried about COVID-19). The exercise frequencies were investigated to measure physical activity level.

Participants were also required to self-report their medical histories, including parities, delivery gestational age, pregestational weight, weight before labor, and disease during pregnancy. Women who could not accurately remember this information were encouraged to check the data with the help of doctors. If an individual had been diagnosed with any diseases during pregnancy, including diabetes, dyslipidemia, or hypothyroidism, she was defined as having a metabolic disease history in data analyses. Pregestational body mass index (BMI) was calculated as pregestational weight (kg) divided by square of height (m^2^). Based on the pregestational BMI, the GWG were categorized as optimal GWG, low GWG, and excess GWG according to Institute of Medicine guidelines (US) (Supplementary Table S1) [15], and women who had GWG lower or higher than the recommended values were classified as low or excess GWG, respectively.

EE was evaluated based on the Chinese version of the Dutch Eating Behavior Questionnaire (DEBQ-C) [16]. The DEBQ-C includes three domains: EE, external eating, and restrained eating. Only the EE domain, consisting of 13 items, was used in the current survey, with regards to the emotions of anxiety, irritation, boredom, depression, loneliness, disappointment, anger, fear, etc. A Likert scale was used to investigate the frequencies of occurrence of the above EE during the COVID-19 pandemic. The frequency was described as “never”, “rarely”, “sometimes”, “often”, or “always” and scored from 1 to 5, respectively. The total score of EE could range from 13 to 65, and a higher score means a higher tendency of EE. In the current study, the EE score was divided into categories by 10 scores. As the proportion of participants whose EE score > 43 was low, those who scored between 33 and 65 were combined into one group, and the final EE score categories were 13–22, 23–32, and 33–65.

For measuring the dietary intake, participants were asked whether the following 12 food groups had quantitative changes (stayed the same, increased, or decreased) during the third trimester (the same time of the lockdown/self-isolation policy implemented in China): (1) cereals; (2) roots and tubers; (3) vegetables; (4) fruits; (5) meat, poultry, and offal; (6) eggs; (7) fish and seafood; (8) pulses, legumes, and nuts; (9) dairy products; (10) oils and fats; (11) sugar and honey; and (12) miscellaneous, such as beverages, snacks, and condiments.

### 2.3. Ethics

The questionnaire was filled in anonymously. Informed consent is required to click to confirm participants’ willingness to participate voluntarily prior to the survey.

### 2.4. Statistics

**Figure 1:**
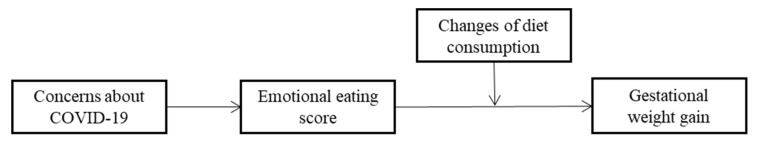
Hypothesis of how emotional eating impacts gestational weight gain.

Continuous variables were presented as means and standard deviations (SDs), and differences across EE score categories were compared with ANOVA. For categorical variables, percentages and Chi-square tests were used. Based on previous studies, we hypothesized that concerns about COVID-19 would promote participants’ EE behavior and would subsequently affect GWG mediation by changes in dietary behaviors (Figure 1). To test the model, ordinal logistic regression models were conducted to estimate the association between concerns about COVID-19 and EE score category. Multinomial logistic regression models were used to investigate whether EE score affects the changes of diet behavior (stayed the same, increased, or decreased) and GWG of participants (optimal, low, or excess). All the models were adjusted by region (northern China, southern China except Wuhan, or Wuhan) and exercise frequency per week (once or less, 2–3, 4–6, or every day).

Subgroup analyses were conducted separately in participants living in Wuhan or other areas. Sensitivity analyses were conducted by additional adjustment for pregestational BMI and metabolic disease history. All statistics were conducted with R 4.0.1. Ordinal logistic regress models and multinomial logistic regression models were conducted with R package MASS and nnet [17], respectively. All *p* values were two-sided, and statistical significance was defined as *p* < 0.05.

## 3. Results

### 3.1. EE Score

Table 1 presents the sociodemographic characteristics of participants across EE score categories. The average EE score of participants was 26.5 ± 8.3. Compared with women living in northern China, those residing in southern China (except Wuhan) and Wuhan had a greater proportion of high EE scores. Women with a higher exercise frequency had a lower EE score. No significant EE score differences were observed among women of different ages, gestational delivery ages, pregestational BMI, education level, number of confirmed COVID-19 cases in the living area, residency, income, parities, or metabolic disease history.

### 3.2. Changes in Food Consumption during COVID-19

**Figure 2:**
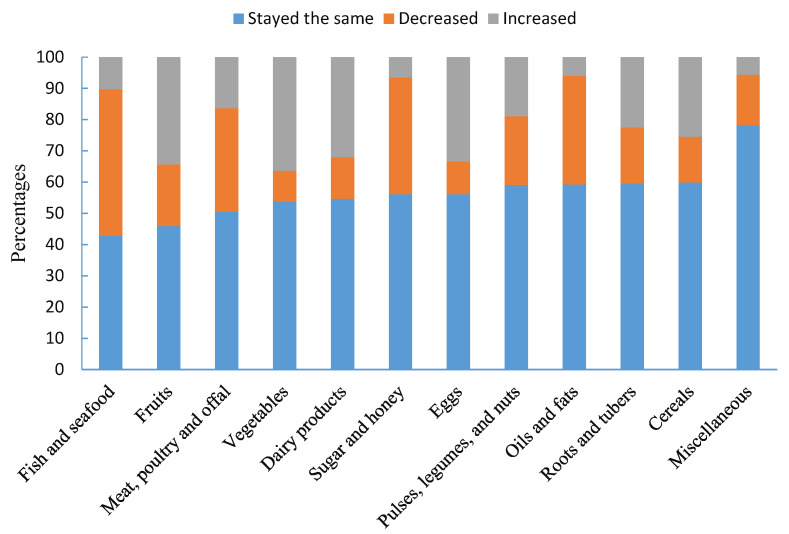
Changes of food consumption in participants (*N* = 640).

In 10 out of the 12 food groups, more than half of the women reported that their consumption during the COVID-19 pandemic stayed the same as before. The top five food groups that participants reported that had decreased consumption were (1) fish and seafood; (2) sugar and honey; (3) oils and fats; (4) meat, poultry, and offal; and (5) pulses, legumes, and nuts. The top five food groups that participants reported that increased in consumption were (1) vegetables; (2) fruits; (3) eggs; (4) dairy products; and (5) cereals (Figure 2).

### 3.3. Concerns About COVID-19 and Its Association with EE 

Results showed that 5.6% of participants reported knowing someone confirmed with COVID-19 and 33.0% of participants severely worried about COVID-19 (concern rank > 8). Table 2 shows that a higher concern about COVID-19 was associated with a higher EE score. Knowing someone confirmed with COVID-19 did not increase the EE score. In the subgroup analysis by living region, the association between COVID-19 concerns and EE score in areas except Wuhan was consistent with the combined population but not in participants living in Wuhan.

### 3.4. EE Score and Its Association with the Changes in Food Consumption

After adjusting for living region and exercise frequency, a higher EE score was associated with increased consumption of cereals and oil and inversely associated with fish consumption (Table 3). The association did not change appreciably in areas except Wuhan, but no association was observed in Wuhan (Supplementary Table S2).

### 3.5. EE Score and Its Association with GWG

The average GWG in EE score 13–22, 23–32, and 33–65 groups were 14.7 ± 5.8, 14.6 ± 5.3, and 14.9 ± 7.0 kg, respectively. The percentages of participants who had optimal, low, and excess GWG were 42.8%, 23.6%, and 33.6%, respectively. Our results indicated that, compared with women who had optimal GWG, a higher EE score increased the risk of excess GWG in women living in areas except Wuhan (Table 4).

### 3.6. Sensitivity Analyses

In the sensitivity analyses, the association between EE score category and GWG did not change after additional adjustment of pregestational BMI and metabolic disease history. In addition, we found that a moderate EE score might be associated with excess GWG in participants living in Wuhan (Supplementary Table S3).

## 4. Discussion

As one of the most vulnerable groups, the health of pregnant women with COVID-19 infection has aroused great concern worldwide. However, there is limited data in regard to the health effects of COVID-19 pandemic on noninfected pregnant women, which is a much bigger group, and who also experienced great lifestyle changes during lockdown time. Our study firstly reported the EE that exists in Chinese pregnant women and it associated with dietary intake and pregnant outcomes.

In this study, the mean score of EE was 26.5 ± 8.3, which was similar to a previous study (before COVID-19) performed in women with higher EE tendency, such as America women with addictive behaviors (26.64 ± 11.89) [18]. There was a total of 20% of participants with an EE score over 32. People living in Wuhan and southern part of China had higher EE scores, which may contribute to a more severe COVID-19 situation in these places [14]. Women who have more physical activities were associated with a lower EE score. Not surprisingly, exercise is considered as one of the most effective ways of regulating mood, [19] and it works as a treatment which could help people to curb EE [20]. In addition, we infer that this inverse association may also be explained by less life restrictions that could both provide a more feasible environment for exercise and could lead to less worry.

The current study reported that women had strong concerns on COVID-19 pandemic, with over 30% of participants rating their concerns [8]. In addition, we observed that the concerns were positively associated with EE tendency; as the psychologist warned, concerns on COVID-19 may trigger the occurrence of EE [11]. To explore the pathway between EE and GWG, firstly, we examined whether EE was related with dietary changes during lockdown time. We observed a direct association between EE and higher cereal and oil food intake. This phenomenon is typical in emotional eating, which usually results in excessive intake of high energy dense food [9]. Our finding was also consistent with studies conducted in Poland and Italia [21,22], which reported that people during lockdown time tend to snack more; however, the potential reasons were not mentioned in these two studies. As demonstrated in a previous study, the reinforcing/preference properties of palatable food are mediated by the mesostriatal dopamine system, which could produce addictive-like DAergic adaptations in the striatum [23]. The increasing consumption of foods with high hedonic value usually results in excess weight gain and increased risks of chronic conditions such as cardiovascular diseases and obesity [24,25,26]. Another finding in the current study deserving our utmost attention is that we observed a decreased consumption of fish in the studied population, especially in women with a higher EE score. Although our previous study conducted in Chinese adults during COVID-19 pandemic indicated that there was a generally good food accessibility and that most food could be accessed via traditional in-person grocery shopping or online delivery, indeed there was an insufficient intake of fish during lockdown time, especially in the regions severely affected by disease where food mostly depended on government/community distribution [27]. On the contrary, studies conducted before COVID-19 revealed that usually there is a rising consumption of most foods including fish in the third trimester [28], which could greatly meet the increasing nutritional need in late pregnancy [29]. Fish could provide high-quality protein, unsaturated fatty acid, and minerals. On one the hand, these nutrients could ensure a more optimal nutritional status for a well-functioning immune system to protect against viral infections [30]. On the other hand, these nutrients are also crucial for fetus growth and development [31]. In this study, unfortunately, we could not estimate the nutrient intake and did not obtain blood samples, so whether nutrients deficiency already occurred in Chinese pregnant women could not be measured. However, the associations between emotional eating, dietary intake, and pregnant outcomes were evaluated.

With a self-reported pregestational weight and weight before labor, the GWG was calculated. Although there is a dietary pattern changed during pandemic, the average GWG in the studied group seems in the normal range and the excess GWG is similar to a study reported pre-COVID-19 pandemic [32]. Although, the effects of EE on weight gain and obesity were well documented [30,33], there is a dearth of research focus on examining potentially modifiable psychosocial predictors of excess GWG. One America study conducted in 2018 indicated the relation between emotional cues and excess GWG and revealed a tendency to eat high fat food being the mediator [34]. In current analyses including sensitive analysis control of pre-BMI and gestational metabolic disease shows that a higher EE score was associated with a higher rate of excess GWG in the population except the ones from Wuhan. Combined with findings on the association between EE and higher cereal and oil intake, we support the hypothesis of “high energy dense foods” cravings mediating the relationship between EE and excess GWG. In a sensitive analysis, we also reported that only a moderate EE score was found associated with excess GWG among Wuhan population; however the interpretation is limited by the sample size. In the case of food, access in Wuhan during lockdown period was highly dependent on food distribution by the government or community; thus, food craving behaviors may not be as significant. However, it is worth noting that women in Wuhan had a higher EE score and more concerns on COVID-19, so physiological intervention is more urgent to this population.

In addition, as documented by a previous study, EE could also act as the restriction of food intake [9]. However, because the emotional eating domain in DEBQ-C we used in this survey only focus on the urges to eat [15], whether EE could cause food restriction and subsequently impose on pregnant health could not be measured. Current data shows that some women indeed had a decreased intake of fish, sugar, oil, meat, and pulse; thus, the mediation model of whether EE occurrence in disease outbreak could be through decreasing consumption of certain food, impacting pregnant health, needs to be urgently explored.

### Limitation

This study is based on one online survey, the participants voluntarily enrolled in this study, and selection bias cannot be ignored. The women who did not respond to this survey may pay less attention to their health and could lead to an adverse pregnant outcome. For methodology, due to unavailability of Chinese guidelines, US guidelines were used in this study to define optimal, low, and excess GWG, which may not fully differentiate the potential risk of GWG in Chinese women. Another concern in the current study is that a series of questions regarding the changes of consumption of 12 food items during the COVID-19 pandemic were used to measure the dietary intake status for its convenience. Based on this methodology, we could not measure the food intake amount and could not estimate the level of nutrients sufficiency or deficiency. In addition, this study was based on a retrospective design and the data of GWG were based on self-report. However, all of our participants were within 7 days of labor and could check these data with her doctor at a charge, so recall bias on weight may not be significant.

## 5. Conclusions

This study revealed that EE occurred in a proportional number of pregnant women during the COVID-19 pandemic and that women living in a severely affected areas who strong worried about the pandemic and who had lower physical activity levels had a higher tendency of EE. Mediated by craving certain food, EE was associated with excess GWG. This study will allow us to develop recommendations on how to manage pregnancy health in noninfected pregnant group in the COVID-19 pandemic and in the case of repeated epidemic emergencies. Based on the findings from the current study, we have proposed the following recommendations: (1) psychological services and education, such as psychological counseling during regular pregnancy check or online counseling, are needed to help women cope with and release stress and to manage occasional EE, especially in the areas severely impacted by COVID-19 and the lockdown policy; (2) nutrition education and intervention are essential to encourage a more balanced diet in pregnancy; and (3) more research is urgently demanded to identify potential nutritional concerns in noninfected pregnant women to provide corresponding strategies.

## Figures and Tables

**Table 1:** Sociodemographic of participants among women with different emotional eating score category.

	Emotional Eating Score Categories a			
	13–22	23–32	33–65	*p*
Number of participants	186	321	133	
Age (year)	29.2 ± 4.6	29.4 ± 4.5	29.8 ± 3.8	0.447
Delivery gestational age (week)	39.5 ± 1.0	39.4 ± 1.2	39.3 ± 0.9	0.339
Pregestational body mass index (kg/m^2^)	21.5 ± 3.2	21.1 ± 2.8	21.5 ± 3.5	0.275
Education level				
Lower middle school or below	12.4	11.2	9.0	0.853
Middle school	19.9	17.8	18.8	
College or above	67.7	71.0	72.2	
Living region				
Northern China	56.5	49.8	35.3	0.002
Southern China (except Wuhan)	36.5	37.1	49.6	
Wuhan	7.0	13.1	15.1	
Number of confirmed COVID-19 cases in living areas				
500 or below	55.4	51.7	42.9	0.081
over 500	44.6	48.3	57.1	
Residency				
Urban	72.0	64.2	72.9	0.080
others	28.0	35.8	27.1	
Monthly household income per person (RMB) ^b^				
Below 5000	24.0	35.1	27.8	0.084
5000–10,000	39.5	29.4	34.9	
10,000 or above	36.5	35.5	37.3	
Parities				
First birth	55.9	57.6	53.4	0.704
Others	44.1	42.4	46.6	
Exercise frequency (times per week)				
Once or lower	13.4	19.6	18.0	0.014
2–3	28.5	25.2	28.6	
3–4	7.0	13.4	18.0	
Everyday	51.1	41.7	35.3	
Metabolic disease history				
No	78.0	81.0	82.7	0.541
Yes	22.0	19.0	17.3	

Continuous variables were presented as means ± SDs, and differences across groups were tested with ANOVA; for categorical variables, percentages and Chi-square tests were used. **^a^** The emotional eating score was divided into categories by each ten score. As the proportion of participants who scored high in EE score was low, those who scored between 33 to 65 were combined into a group. **^b^** Forty-eight missing values.

**Table 2:** Concerns about the novel 2019 coronavirus disease (COVID-19) and emotional eating score category.

	Number of Participants	Crude	Adjusted
		OR (95% CI)	OR (95% CI)
**Combined**			
Knowing someone who confirmed with COVID-19			
No	604	Ref.	Ref.
Yes	36	2.12 (1.15, 3.91)	1.81 (0.92, 3.56) ^a^
Concerns on COVID-19			
7 or below	428	Ref.	Ref.
8 or above	212	1.48 (1.08, 2.03)	1.51 (1.10, 2.07) ^a^
**Areas except Wuhan**			
Knowing someone who confirmed with COVID-19			
No	553	Ref.	Ref.
Yes	12	1.34 (0.50, 3.59)	1.43 (0.52, 3.93) ^a^
Concerns on COVID-19			
7 or below	382	Ref.	Ref.
8 or above	183	1.43 (1.02, 2.00)	1.47 (1.05, 2.06) ^a^
**Wuhan**			
Knowing someone who confirmed with COVID-19			
No	51	Ref.	Ref.
Yes	24	2.38 (0.92, 6.34)	2.54 (0.98, 6.87) ^b^
Concerns on COVID-19			
7 or below	46	Ref.	Ref.
8 or above	29	1.75 (0.71, 4.44)	1.57 (0.61, 4.12) ^b^

Ordinal logistic regression models were conducted with the lowest score group of emotional eating score category as the comparison group. **^a^** Models were adjusted for living region and exercise frequency. **^b^** Models were adjusted for exercise frequency.

**Table 3:** Association of emotional eating score and changes of food consumption.

		Increased Consumption			Decreased Consumption		
		Emotional Eating Score Categories OR (95% CI)			Emotional Eating Score Categories OR (95% CI)		
Numbers of participants		13–22	23–32	32–65	13–22	23–32	32–65
Cereals	Crude	Ref.	1.46 (0.93, 2.30)	2.16 (1.27, 3.67)	Ref.	1.04 (0.62, 1.76)	1.26 (0.66, 2.41)
	Adjusted ^a^	Ref.	1.48 (0.94, 2.34)	2.22 (1.29, 3.82)	Ref.	0.95 (0.55, 1.61)	1.17 (0.60, 2.28)
Roots and tubers	Crude	Ref.	1.08 (0.69, 1.69)	1.40 (0.81, 2.39)	Ref.	1.40 (0.85, 2.32)	1.37 (0.74, 2.54)
	Adjusted ^a^	Ref.	1.07 (0.68, 1.70)	1.44 (0.82, 2.50)	Ref.	1.32 (0.79, 2.19)	1.28 (0.68, 2.42)
Vegetables	Crude	Ref.	0.90 (0.62, 1.33)	0.90 (0.56, 1.46)	Ref.	1.17 (0.60, 2.27)	1.60 (0.75, 3.42)
	Adjusted ^a^	Ref.	0.92 (0.62, 1.35)	0.94 (0.57, 1.54)	Ref.	1.00 (0.50, 1.98)	1.37 (0.62, 3.02)
Fruits	Crude	Ref.	0.92 (0.62, 1.38)	1.10 (0.67, 1.81)	Ref.	1.38 (0.83, 2.30)	1.64 (0.89, 3.03)
	Adjusted ^a^	Ref.	0.96 (0.64, 1.44)	1.20 (0.72, 2.00)	Ref.	1.25 (0.74, 2.10)	1.45 (0.77, 2.73)
Meat, poultry and offal	Crude	Ref.	1.17 (0.69, 1.97)	1.62 (0.87, 3.02)	Ref.	1.27 (0.84, 1.91)	1.52 (0.92, 2.52)
	Adjusted ^a^	Ref.	1.20 (0.71, 2.03)	1.71 (0.90, 3.23)	Ref.	1.20 (0.79, 1.82)	1.45 (0.86, 2.43)
Fish and seafood	Crude	Ref.	0.78 (0.42, 1.47)	1.67 (0.83, 3.36)	Ref.	1.91 (1.30, 2.82)	2.02 (1.24, 3.28)
	Adjusted ^a^	Ref.	0.76 (0.40, 1.43)	1.70 (0.83, 3.50)	Ref.	1.79 (1.20, 2.66)	1.88 (1.14, 3.11)
Eggs	Crude	Ref.	0.81 (0.54, 1.20)	1.04 (0.65, 1.68)	Ref.	0.84 (0.47, 1.51)	0.62 (0.28, 1.38)
	Adjusted ^a^	Ref.	0.82 (0.55, 1.23)	1.06 (0.65, 1.73)	Ref.	0.80 (0.44, 1.46)	0.60 (0.26, 1.38)
Dairy products	Crude	Ref.	0.71 (0.47, 1.05)	0.93(0.57, 1.51)	Ref.	0.94 (0.54, 1.64)	0.96 (0.48, 1.92)
	Adjusted ^a^	Ref.	0.75 (0.50, 1.12)	1.06 (0.64, 1.75)	Ref.	0.86 (0.48, 1.51)	0.83 (0.40, 1.70)
Pulses, legumes, and nuts	Crude	Ref.	1.18 (0.73, 1.93)	1.49 (0.83, 2.68)	Ref.	1.04 (0.66, 1.63)	1.28 (0.74, 2.22)
	Adjusted ^a^	Ref.	1.21 (0.73, 1.99)	1.60 (0.88, 2.92)	Ref.	0.94 (0.59, 1.49)	1.15 (0.65, 2.04)
Oils and fats	Crude	Ref.	2.15 (0.84, 5.47)	2.65 (0.94, 7.45)	Ref.	1.06 (0.72, 1.55)	0.94 (0.58, 1.52)
	Adjusted ^a^	Ref.	2.29 (0.89, 5.87)	3.03 (1.06, 8.69)	Ref.	1.00 (0.68, 1.48)	0.86 (0.52, 1.41)
Sugar and honey	Crude	Ref.	1.17 (0.53, 2.62)	2.01(0.84, 4.84)	Ref.	1.16 (0.79, 1.70)	1.15 (0.72, 1.85)
	Adjusted ^a^	Ref.	1.14 (0.51, 2.57)	1.95 (0.80, 4.79)	Ref.	1.14 (0.77, 1.68)	1.10 (0.68, 1.79)
Miscellaneous ^b^	Crude	Ref.	1.18 (0.52, 2.70)	1.51 (0.58, 3.92)	Ref.	1.08 (0.65, 1.78)	1.34 (0.74, 2.44)
	Adjusted ^a^	Ref.	1.22 (0.53, 2.82)	1.77 (0.66, 4.73)	Ref.	0.96 (0.57, 1.61)	1.19 (0.64, 2.22)

Multinomial logistic regression models were conducted, with participants whose consumption stayed the same as the comparison group. **^a^** Models were adjusted for living region and exercise frequency. **^b^** Miscellaneous includes beverage, snacks, and condiments.

**Table 4:** Association of emotional eating score and gestational weight gain.

		Emotional Eating Score Categories OR (95% CI)		
		13–22	23–32	33–65
**Low weight gain**				
Combined	Crude	Ref.	1.35 (0.85, 2.14)	1.38 (0.78, 2.46)
	Adjusted ^a^	Ref.	1.34 (0.84, 2.15)	1.40 (0.78, 2.53)
Areas except Wuhan	Crude	Ref.	1.18 (0.72, 1.93)	1.55 (0.83, 2.88)
	Adjusted ^a^	Ref.	1.19 (0.72, 1.95)	1.61 (0.86, 3.04)
Wuhan	Crude	Ref.	3.33 (0.72, 15.37)	0.89 (0.16, 5.08)
	Adjusted ^b^	Ref.	3.52 (0.74, 16.83)	0.87 (0.15, 5.16)
**Excess weight gain**				
Combined	Crude	Ref.	1.34 (0.88, 2.04)	1.51 (0.91, 2.53)
	Adjusted ^a^	Ref.	1.40 (0.92, 2.14)	1.69 (1.00, 2.85)
Areas except Wuhan	Crude	Ref.	1.20 (0.78, 1.86)	1.71 (0.99, 2.95)
	Adjusted ^a^	Ref.	1.24 (0.80, 1.93)	1.90 (1.08, 3.32)
Wuhan	Crude	Ref.	5.00 (0.89, 28.07)	1.33 (0.20, 9.08)
	Adjusted ^b^	Ref.	4.93 (0.85, 28.68)	1.19 (0.17, 8.30)

Multinomial logistic regression models were conducted, with women having optimal gestational weight gain as the comparison group. **^a^** Models were adjusted for living region and exercise frequency. **^b^** Models were adjusted for exercise frequency.

## Supplementary Materials

The following are available online at https://www.mdpi.com/2072-6643/12/8/2250/s1, Table S1: Recommendations for total weight gain during pregnancy by the pre-pregnancy body mass index, Table S2: Subgroup analysis of the association of emotional eating score and changes of food consumption, Table S3: Sensitivity analysis of association of emotional eating score and gestational weight gain.
