# Supplementary material for: Emotional Eating in Pregnant Women during the COVID-19 Pandemic and Its Association with Dietary Intake and Gestational Weight Gain
Source: Nutrients. 2020 Jul 28;12(8):2250. doi: 10.3390/nu12082250 (PMC7468999; doi:10.3390/nu12082250)
Supplement: Supplementary file 1 [file nutrients-12-02250-s001.pdf]

Supplementary Table S1. Recommendations for total weight gain during pregnancy by the pre-pregnancy body mass index [a].

Pre-pregnancy body mass index (kg/m^2)	Total gestational weight gain (kg)
Underweight (<18.5)	12.5–18.0
Normal weight (18.5–24.9)	11.5–16.0
Overweight (25.0–29.9)	7.0–11.5
Obese (≥30.0)	5.0–9.0

[a] Institute of Medicine (US) and National Research Council (US) Committee to Reexamine IOM Pregnancy Weight Guidelines. *Weight Gain During Pregnancy: Reexamining the Guidelines*; Rasmussen, K.M., Yaktine, A.L., Eds.; National Academies Press (US): Washington, DC, 2009.

Supplementary Table S2. Subgroup analysis of the association of emotional eating score and changes of food consumption.

		**Areas Except Wuhan** OR (95% CI)[a]			**Wuhan** OR (95% CI)[b]		
		13–22	23–32	32–65	13–22	23–32	32–65
Numbers of participants		173	279	113	13	42	20
**Increased consumption**							
Cereals	Crude	Ref..	1.66 (1.02, 2.69)	2.73 (1.55, 4.81)	Ref.	0.35 (0.08, 1.50)	0.20 (0.03, 1.22)
	Adjusted	Ref..	1.71 (1.05, 2.79)	2.91 (1.63, 5.19)	Ref.	0.30 (0.07, 1.36)	0.17 (0.03, 1.11)
Roots and tubers	Crude	Ref..	1.13 (0.70, 1.84)	1.52 (0.86, 2.71)	Ref.	0.43 (0.10,1.89)	0.42 (0.08, 2.25)
	Adjusted	Ref..	1.17 (0.72, 1.91)	1.68 (0.93, 3.03)	Ref.	0.39 (0.08, 1.78)	0.36 (0.06, 2.08)
Vegetables	Crude	Ref..	0.88 (0.59, 1.31)	0.90 (0.54, 1.49)	Ref.	1.04 (0.24, 4.59)	0.89 (0.16, 5.11)
	Adjusted	Ref..	0.90 (0.60, 1.35)	0.94 (0.56, 1.58)	Ref.	0.92 (0.20, 4.30)	0.81 (0.13, 4.90)
Fruits	Crude	Ref..	0.97 (0.64, 1.47)	1.08 (0.64, 1.81)	Ref.	0.59 (0.11, 3.29)	1.33 (0.19, 9.31)
	Adjusted	Ref..	0.99 (0.65, 1.50)	1.15 (0.67, 1.96)	Ref.	0.52 (0.09, 3.13)	1.12 (0.15, 8.42)
Meat, poultry and offal	Crude	Ref..	1.21 (0.70, 2.08)	1.68 (0.88, 3.24)	Ref.	0.62 (0.09, 4.49)	0.86 (0.10, 7.51)
	Adjusted	Ref..	1.25 (0.72, 2.16)	1.80 (0.92, 3.51)	Ref.	0.60 (0.08, 4.54)	0.86 (0.09, 8.22)
Fish and seafood	Crude	Ref..	0.84 (0.43, 1.61)	1.61 (0.77, 3.37)	Ref.	0.20 (0.02, 2.39)	1.00 (0.08, 12.56)
	Adjusted	Ref..	0.81 (0.42, 1.58)	1.67 (0.78, 3.59)	Ref.	0.15 (0.01, 2.08)	0.67 (0.05, 9.90)
Eggs	Crude	Ref..	0.80 (0.53, 1.21)	1.11 (0.67, 1.83)	Ref.	0.76 (0.20, 2.98)	0.60 (0.12, 2.97)
	Adjusted	Ref..	0.81 (0.53, 1.24)	1.13 (0.67, 1.90)	Ref.	0.76 (0.18, 3.13)	0.57 (0.11, 3.01)
Dairy products	Crude	Ref..	0.77 (0.51, 1.16)	0.94 (0.57, 1.57)	Ref.	0.29 (0.07, 1.31)	0.71 (0.13, 3.87)
	Adjusted	Ref..	0.80 (0.53, 1.22)	1.07 (0.63, 1.80)	Ref.	0.23 (0.04, 1.17)	0.64 (0.10, 3.95)

Pulses, legumes, and nuts	Crude	Ref..	1.29 (0.77, 2.15)	1.60 (0.86, 2.96)	Ref.	0.30 (0.05, 1.89)	0.43 (0.05, 3.48)
	Adjusted	Ref..	1.33 (0.79, 2.24)	1.76 (0.94, 3.31)	Ref.	0.28 (0.04, 1.86)	0.41 (0.05, 3.56)
Oils and fats[c]	Crude	Ref..	2.09 (0.81, 5.40)	2.63 (0.92, 7.57)	-	-	-
	Adjusted	Ref..	2.20 (0.85, 5.71)	2.96 (1.01, 8.67)	-	-	-
Sugar and honey	Crude	Ref..	1.02 (0.42, 2.44)	2.20 (0.88, 5.52)	Ref.	1.58 (0.15, 16.31)	0.75 (0.04, 14.58)
	Adjusted	Ref..	1.04 (0.43, 2.50)	2.26 (0.88, 5.79)	Ref.	1.37 (0.13, 14.68)	0.61 (0.03, 12.23)
Miscellaneous	Crude	Ref..	1.17 (0.48, 2.82)	1.56 (0.56, 4.29)	Ref.	1.00 (0.09, 10.87)	1.00 (0.05, 18.58)
	Adjusted	Ref..	1.25 (0.51, 3.05)	1.97 (0.70, 5.55)	Ref.	0.79 (0.07, 9.07)	0.82 (0.04, 16.04)
**Decreased consumption**							
Cereals	Crude	Ref..	0.99 (0.57, 1.74)	1.04 (0.50, 2.18)	Ref.	0.63 (0.12, 3.39)	0.93 (0.16, 5.54)
	Adjusted	Ref..	0.97 (0.55, 1.71)	1.04 (0.49, 2.22)	Ref.	0.42 (0.07, 2.60)	0.62 (0.09, 4.25)
Roots and tubers	Crude	Ref..	1.30 (0.76, 2.22)	1.17 (0.59, 2.33)	Ref.	1.10 (0.21, 5.75)	1.17 (0.19, 7.12)
	Adjusted	Ref..	1.30 (0.76, 2.24)	1.18 (0.59, 2.39)	Ref.	0.84 (0.15, 4.89)	0.84 (0.12, 5.67)
Vegetables	Crude	Ref..	1.20 (0.56, 2.58)	1.22 (0.48, 3.12)	Ref.	0.62 (0.13, 2.91)	1.43 (0.27, 7.52)
	Adjusted	Ref..	1.17 (0.54, 2.53)	1.19 (0.46, 3.10)	Ref.	0.39 (0.07, 2.24)	0.97 (0.15, 6.22)
Fruits	Crude	Ref..	1.44 (0.82, 2.53)	1.45 (0.73, 2.90)	Ref.	0.59 (0.14, 2.48)	1.33 (0.25, 7.01)
	Adjusted	Ref..	1.41 (0.80, 2.50)	1.39 (0.69, 2.84)	Ref.	0.50 (0.11, 2.22)	1.12 (0.20, 6.27)
Meat, poultry and offal	Crude	Ref.	1.25 (0.81, 1.93)	1.51 (0.88, 2.59)	Ref.	0.75 (0.19, 3.01)	0.82 (0.17, 3.90)
	Adjusted	Ref.	1.24 (0.80, 1.93)	1.50 (0.86, 2.61)	Ref.	0.53 (0.11, 2.49)	0.54 (0.10, 3.01)
Fish and seafood	Crude	Ref.	1.93 (1.28, 2.91)	1.93 (1.15, 3.23)	Ref.	0.67 (0.12, 3.62)	1.04 (0.14, 7.48)
	Adjusted	Ref.	1.91 (1.26, 2.89)	1.92 (1.13, 3.25)	Ref.	0.48 (0.08, 2.98)	0.66 (0.08, 5.52)
Eggs	Crude	Ref.	0.82 (0.44, 1.53)	0.37 (0.13, 1.03)	Ref.	0.82 (0.13, 5.14)	1.50 (0.22, 10.3)
	Adjusted	Ref.	0.84 (0.45, 1.57)	0.40 (0.14, 1.12)	Ref.	0.50 (0.07, 3.85)	0.99 (0.12, 8.53)
Dairy products	Crude	Ref.	0.88 (0.48, 1.61)	0.61 (0.26, 1.42)	Ref.	0.76 (0.15, 3.78)	1.90 (0.33, 11.01)
	Adjusted	Ref.	0.88 (0.48, 1.62)	0.58 (0.25, 1.38)	Ref.	0.62 (0.12, 3.24)	1.54 (0.26, 9.30)
Pulses, legumes, and nuts	Crude	Ref.	1.05 (0.64, 1.73)	1.15 (0.62, 2.14)	Ref.	0.34 (0.08, 1.54)	0.61 (0.12, 3.23)
	Adjusted	Ref.	1.05 (0.63, 1.72)	1.15 (0.61, 2.18)	Ref.	0.27 (0.05, 1.32)	0.46 (0.08, 2.60)

Oils and fats	Crude	Ref.	1.08 (0.72, 1.62)	0.93 (0.55, 1.56)	Ref.	0.57 (0.16, 2.03)	0.56 (0.13, 2.36)
	Adjusted	Ref.	1.08 (0.71, 1.63)	0.88 (0.52, 1.51)	Ref.	0.44 (0.11, 1.70)	0.43 (0.09, 1.96)
Sugar and honey	Crude	Ref.	1.16 (0.77, 1.73)	1.05 (0.63, 1.75)	Ref.	0.95 (0.26, 3.48)	1.38 (0.32, 5.88)
	Adjusted	Ref.	1.17 (0.78, 1.76)	1.03 (0.61, 1.74)	Ref.	0.87 (0.23, 3.29)	1.23 (0.28, 5.44)
Miscellaneous [d]	Crude	Ref.	0.97 (0.56, 1.67)	0.93 (0.47, 1.87)	Ref.	1.33 (0.31, 5.82)	3.33 (0.68, 16.3)
	Adjusted	Ref.	0.95 (0.54, 1.64)	0.90 (0.44, 1.84)	Ref.	1.12 (0.24, 5.17)	3.11 (0.59, 16.34)

Multinomial logistic regression models were conducted, with participants whose consumption stayed the same as the comparison group. [a] Adjusted models were adjusted for living region and exercise frequency. [b] Adjusted models were adjusted for and exercise frequency. [c] As the number of participants living in Wuhan is small and the proportion of participants reported increased oil consumption was low (4%), ORs and CIs could not be assessed precisely. [d] Miscellaneous includes beverage, snacks and condiments.

Supplementary Table S3. Sensitivity analysis of association of emotional eating score and gestational weight gain.

	**Emotional Eating Score Categories OR (95% CI)**		
	**13–22**	**23–32**	**33–65**
**Low weight gain**			
Combined [a]	Ref.	1.35 (0.84, 2.16)	1.40 (0.77, 2.53)
Areas except Wuhan [a]	Ref.	1.20 (0.73, 1.97)	1.63 (0.86, 3.09)
Wuhan [b]	Ref.	4.74 (0.93, 24.00)	1.06 (0.17, 6.49)
**Excess weight gain**			
Combined	Ref.	1.43 (0.94, 2.20)	1.67 (0.98, 2.83)
Areas except Wuhan	Ref.	1.29 (0.83, 2.01)	1.89 (1.08, 3.34)
Wuhan	Ref.	7.60 (1.22, 47.57)	1.67 (0.23, 12.20)

Multinomial logistic regression models were conducted, with women had optimal gestational weight gain as the comparison group. [a] Models were adjusted for living region, exercise frequency, pregestational body mass index, and gestation weight gain. [b] Models were adjusted for exercise frequency, pregestational body mass index, and gestation weight gain.
